# Evaluating the accuracy of automated processing of child and adult language production in preschool classrooms

**DOI:** 10.3389/fpsyg.2024.1322665

**Published:** 2024-06-26

**Authors:** G. Logan Pelfrey, Laura M. Justice, Hugo Gonzalez Villasanti, Tiffany J. Foster

**Affiliations:** ^1^Crane Center for Early Childhood Research and Policy, The Ohio State University, Columbus, OH, United States; ^2^Department of Mechanical Engineering, University of Michigan, Ann Arbor, MI, United States

**Keywords:** speech processing, early childhood language environments, preschool, automated sensing, objective measurement

## Abstract

Young children's language and social development is influenced by the linguistic environment of their classrooms, including their interactions with teachers and peers. Measurement of the classroom linguistic environment typically relies on observational methods, often providing limited 'snapshots' of children's interactions, from which broad generalizations are made. Recent technological advances, including artificial intelligence, provide opportunities to capture children's interactions using continuous recordings representing much longer durations of time. The goal of the present study was to evaluate the accuracy of the Interaction Detection in Early Childhood Settings (IDEAS) system on 13 automated indices of language output using recordings collected from 19 children and three teachers over two weeks in an urban preschool classroom. The accuracy of language outputs processed via IDEAS were compared to ground truth via linear correlations and median absolute relative error. Findings indicate high correlations between IDEAS and ground truth data on measures of teacher and child speech, and relatively low error rates on the majority of IDEAS language output measures. Study findings indicate that IDEAS may provide a useful measurement tool for advancing knowledge about children's classroom experiences and their role in shaping development.

## Accuracy of automated processing of child and adult language production in preschool classrooms

Approximately four of five children aged 3–5 years across the globe are enrolled in some form of out-of-home preschool program (Organization for Economic Co-operation and Development, 2022), and evidence shows that features of these programs are associated with young children's cognitive and social development (Umek, 2011; Justice et al., 2018; Foster et al., 2020). Of relevance to the present study, children's exposure to teacher and peer linguistic input within preschool classrooms influences their growth in language skills over time (Huttenlocher et al., 2002; Justice et al., 2018). For instance, studies find significant relations between the quality of teacher-child linguistic interactions and children's development of receptive and expressive language skills (Gest et al., 2006; Dickinson et al., 2008; Douglas et al., 2021; Yang et al., 2021), and a number of studies show that exposure to peer language in the preschool classroom is associated with young children's language development (Justice et al., 2014; Chen et al., 2020). Given that many children spend considerable hours within preschool classrooms during these early formative years, educational researchers are increasingly investigating the precise manner in which teacher- and peer-linguistic interactions within these classroom settings affect young children's language development (Dickinson et al., 2008; Cabell et al., 2015).

To advance this line of research, in the present study we evaluated the accuracy of the Interaction Detection in Early Academic Settings (IDEAS) system as applied to long-form audio-recordings of children's exposure to peer and teacher talk, as well as their own talk, in a preschool classroom setting. IDEAS is a novel, low-cost sensing system that is designed to automatically process 13 indices of teacher and child talk in early childhood environments (Sun et al., 2023). The accuracy of the proximity component of the system is reported via a separate study (Shehab et al., 2024). By evaluating the accuracy of IDEAS speech indices, which is the focus of the current study, researchers may have a useful tool to supplement traditional observation methods for studying language in preschool classroom settings.

### Traditional methods for studying classroom language environments

Recent research findings have advanced understanding of children's language experiences in preschool settings (Bratsch-Hines et al., 2019; Paatsch et al., 2019; Kurkul et al., 2022). This body of research shows, for instance, that children's language experiences in classrooms settings vary as a function of teacher quality, location in the classroom, or activity (Sawyer et al., 2017; Plummer-Wilson, 2020). As an example, Bratsch-Hines et al.'s (2019) study of 455 preschool children's classroom language experiences found that children's growth in expressive language was positively associated with child-teacher language exchanges and negatively associated with the frequency of large-group activities. However, much of this body of research has relied upon brief, periodic in-person observational research methods, a common method for examining children's early language experiences (d'Apice et al., 2019; Phillips et al., 2019; Burchinal et al., 2021) that presents several limitations.

First, in-person observations are susceptible to observer bias (Hunter, 2020; White et al., 2022), which refers to systematic deviations from the truth that occur due to observer and contextual characteristics (Mahtani et al., 2018). For instance, evidence suggests that an observer's emotional state can bias observation ratings (Floman et al., 2016). Importantly, there is also evidence of gender bias in observer ratings of children, such that gender mismatch between observers and children can lead to higher scores of problematic behaviors (Pellegrini, 2011). In addition, observers' ratings can vary depending on a variety of contextual factors, such as time of day, child grouping configuration (e.g., whole vs. small group), content covered during the observation, and classroom composition (Thorpe et al., 2020).

Second, in-person observations can be prohibitively expensive to implement, as these require on-site (or virtual) human personnel to conduct such observations (Pianta and Hamre, 2009). Consequently, larger-scale studies of children's language experiences often rely on infrequent, brief observations of children, upon which generalizations are drawn (Rankin et al., 2022; Vitiello et al., 2022). For instance, in a study of the effectiveness of teacher-child instructional interactions, Cabell et al. (2013) analyzed data derived from a single observation conducted in each of 314 preschool classrooms. The observations ranged from two to four hours and served as their primary variable of interest, yet these observations represented only 0.003% of children's overall classroom experiences, based on our estimates. Similarly, Sawyer et al. (2017) observed classrooms for one 25-min timepoint in their study of variation in preschool classroom language environments. This observation duration represents ~ <1% of children's classroom experiences throughout the school year (our estimate). With these examples in mind, it is unclear if such studies provide an accurate representation of children's language environments in naturalistic preschool classroom settings.

Third, traditional in-person classroom observations typically involve only one child being observed at a time, and usually for a small portion of the school day (Bratsch-Hines et al., 2019). There is evidence that a range of child characteristics, such as disability status and temperament, relate to the amount of talk to which children are exposed (Rudasill and Rimm-Kaufman, 2009; Irvin et al., 2013; Bergelson et al., 2018b; Chen et al., 2020). For instance, Chen et al. (2020) study of 448 preschool children showed children with disabilities experienced significantly less exposure to peer language resources than their typically developing peers. Such findings raise questions about the validity of researchers' examination of the language experiences of all children in the classroom based on the observation of only one child.

With these limitations in mind, researchers are actively exploring alternative methods to observe preschool classroom environments that overcome the limitations inherent to observational methods (e.g., Bergelson et al., 2018a; Gonzalez Villasanti et al., 2020; Irvin et al., 2021). In particular, sensing technologies are increasingly being used as a means to more broadly capture children's language experiences in preschool settings.

### Sensing technologies for studying classroom language environments

Sensing technologies provide an alternative approach to traditional in-person observations that could address the limitations previously described and help provide more objective representations of young children's language experiences in classroom settings. These sensing technologies typically comprise an audio-recording and/or proximity-tracking device, often used in tandem and worn by participants for the majority of a school day (Irvin et al., 2021; Perry et al., 2022). The system records continuous incoming and outgoing talk, proximity to others in the classroom, and orientation data simultaneously for all children and adults wearing the devices. Such technologies have the potential to provide unprecedented amounts of data on the continuous and oftentimes fleeting interactions that occur among children and adults throughout the school day (Kothalkar et al., 2021), and address several of the limitations of in-person observations. Specifically, sensing technologies can be implemented in the absence of a human observer in the room to provide objective data on children's experiences, thus eliminating observer bias; are very low cost when relying on open-source software; and can capture the language experiences of all children and teachers in a classroom simultaneously. And, as we address in this article, the accuracy of the data generated from sensing technology for several measures such as word, utterance, and conversational turn count are highly correlated with indices calculated via manually timestamped and transcribed observational data.

An emerging body of research indicates that sensing technologies can be used to understand young children's early language environments (see Irvin et al., 2021), and examine relations between features of these environments and children's developmental outcomes (Sangwan et al., 2015; Greenwood et al., 2018; Romeo et al., 2018). To date, the Language ENvironment Analysis (LENA) (Sangwan et al., 2015) and Ubisense systems (Killijian et al., 2016) appear to be the two systems most commonly used to examine children's experiences across a variety of contexts (Gilkerson et al., 2017; Romeo et al., 2018; Messinger et al., 2019; Irvin et al., 2021; Kothalkar et al., 2021; Mitsven et al., 2022). Although primarily designed for use in at-home (LENA) and industrial (Ubisense) environments, research teams are now using these technologies in preschool settings to study language and social network phenomena (e.g., Fasano et al., 2021; Irvin et al., 2021) and have advanced knowledge in the field of early learning in several notable ways.

For instance, Mitsven et al. (2022) used LENA to collect more than 21 h of teacher and preschooler vocalizations in an oral-language preschool classroom to examine the associations between phonemic diversity and language development for children with and without hearing loss. The investigators found that objectively measured phonemic diversity of child vocalizations was a stronger predictor of child language development than hearing status. The authors propose that exposing children with hearing loss to phonemically diverse incoming language and providing them with opportunities for the production of phonemically diverse speech, may further support their development of language skills. As another example, Kothalkar et al. (2021) used a combination of LENA and Ubisense to collect nearly 30 h of preschool classroom recordings to identify the activities and areas that enhance teachers' and children's use of Wh- questions. Teachers use Wh- questions to facilitate exploration, expand children's engagement, and scaffold their learning. Study findings indicated that a significantly higher frequency of Wh- questions occurred in reading areas than science areas. By identifying where Wh- questions happen most frequently, we may support teachers in incorporating these questions into other areas of the classroom to further support children's development and exploration of concepts.

Existing studies using sensing systems in preschool classrooms often focus on adult (teacher) talk directed toward children (Irvin et al., 2013; Soderstrom and Wittebolle, 2013), typically with LENA as the primary tool for speech processing (Wang et al., 2017). While there are strong theoretical reasons for focusing on adult talk (Huttenlocher et al., 1991; Massey, 2004; Gilkerson and Richards, 2009; Irvin et al., 2015), an additional explanation may be driven by the outcome variables LENA provides. Specifically, LENA outputs include the following three core measures—adult word count, child vocalization count, and conversational turns—which are somewhat limited in representing children's classroom language experiences. First, with respect to adult word count, the extant literature makes it clear that other characteristics of adult talk to children influence their language development (Smith and Dickinson, 1994). For instance, a recent study by Dore et al. (2022) showed strong concurrent relations between the syntactic complexity of adult talk and children's expressive and receptive language skills. Second, concerning child vocalization count, LENA provides a somewhat coarse representation of children's own talk. In particular, the LENA system does not make distinctions between child vocalizations and verbalizations, with only the latter representing talk. In addition, by providing only a frequency count of a child's vocalizations, LENA does not provide a more nuanced representation of a child's language production in terms of semantic, syntactic, and morphologic characteristics. Finally, LENA does not capture talk spoken by other children, as it was not created for use in classroom settings. However, recent studies show that children's language growth in preschool classrooms is influenced by the language input they receive from peers, and that these relations operate independently from the influence of teachers' talk (Chen et al., 2020).

There is evidence that sensing systems, such as LENA, can be used in preschool settings to model children's language experiences over time in ways that expand upon traditional observation techniques. Perry et al. (2018) used LENA to capture 680 h of the language experiences of 13 children collected each week in a preschool classroom over an academic year. This study found that both peer vocalizations and children's conversational turns with teachers were associated with children's language development over the school year (Perry et al., 2018). Whereas this study advances the field by showing the importance of children's exposure to peer and teacher talk over time in classroom settings, there is a need to understand more precisely what aspects of peer and teacher talk influence children's language development in classroom settings. For instance, it seems plausible that exposure to grammatically advanced language from peers or teachers might enhance children's language development (Huttenlocher et al., 2002; Henry and Rickman, 2007; Yeomans-Maldonado et al., 2019), yet the sensing technologies currently used do not capture grammatic elements of language.

Sensing technologies provide promising avenues for capturing children's language experiences in naturalistic preschool classroom environments. However, there are risks to relying solely on technology-based observational data without properly examining the accuracy of these systems, as this could lead to erroneous conclusions. Accordingly, we sought to examine the accuracy of IDEAS in terms of capturing children's language environments with reference to three constructs: (a) teacher talk to a child, (b) peer talk to a child, and (c) child's own talk. Across each of these three constructs, 13 indices of teacher, peer, and child's own talk included number of utterances, number of words, number of verbs, number of auxiliary verbs, number of coordinating conjunctions, number of unique words, number of rare words, number of subordinating conjunctions, number of adjectives, mean length of utterance, type token ratio, conversational turns, and speech duration. This study makes an important contribution to the literature because no other sensing systems of which we are aware used for the study of interactions in naturalistic preschool settings provide these 13 indices, which more broadly represent the complexity of children's language environments.

## Method

### IDEAS feature set

Here we provide an in-depth overview of IDEAS features. IDEAS collects continuous classroom interaction data using Bluetooth beacons for physical proximity and voice recorders for speech activity. The IDEAS system triangulates proximity and recorded speech activity data to understand with whom children are interacting, and the nature of those interactions. Data is then processed via an automated pipeline that utilizes automatic speech recognition and machine learning to provide outputs that include classroom- and child-level measures of language and mutual affiliation.

The hardware used for data capture includes Bluetooth antennas and beacons, wearable audio recorders, and a laptop running the data collection software. IDEAS utilizes an open-source Bluetooth proximity detection software called DirAct, developed by reelyActive (Mundnich et al., 2019). The system employs three to five **Bluetooth** Low Energy (**BLE**) antennas developed by reelyActive and mounted on the wall in the classroom. Children wear BLE beacons with accelerometers that detect nearby beacons (participants) and transmit the exchanged signal strength indicator (RSSI) to the antennas. These antennas, in turn, transmit the RSSI signal to the laptop in real time, where it is stored in a log file. Children wear Sony ICDUX570 voice recorders for the duration of the observation. The system can process any audio files in mp4 format, so that other recorders may be used as well. For instance, the current study was conducted during IDEAS beta testing, during which children were not wearing the standard Sony ICDUX recorder. They wore GoPro cameras with audio recording capability.

**Figure 1:**
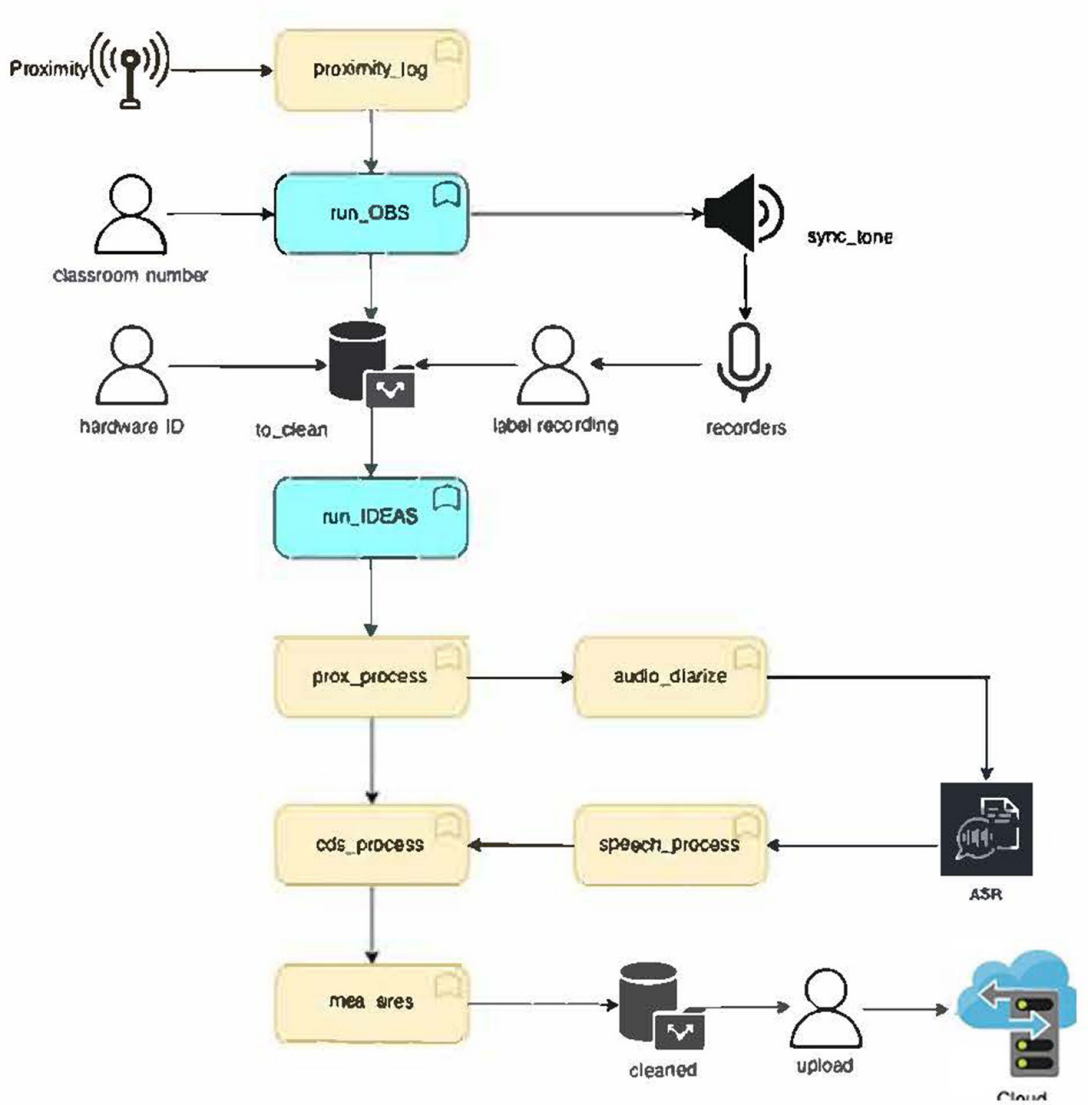
Convergence plots of ERRmedian and correlation values for number of utterances across audio segments.

The IDEAS data pipeline, programmed using the MATLAB platform, consists of the data collection and processing and is represented in the diagram in Figure 1. After turning on beacons and voice recorders, and plugging in the BLE antennas, the observation pipeline starts upon command. This program performs Bluetooth connectivity checks and emits a synchronization tone, which serves to synchronize the Bluetooth and audio data streams in the processing pipeline. At the end of the data collection, the system generates a log file containing the proximity data and the observation's onset, offset, and synchronization signal timestamps. Audio recordings are saved to a memory card in the voice recorder and later uploaded to the laptop for processing.

Once audio recordings are uploaded to the laptop, data is then processed via an automated data processing pipeline. Data processing consists of six stages. The first stage of data processing is segment identification in which the system identifies sections of audio containing valid speech data. During this stage, the proximity data are filtered such that speech data exchanged while participants are within 1.5 m (RSSI = −74 dB) is selected for further processing. The program automatically detects the synchronization tone in the audio recordings with a band-pass filter, and then identifies valid segments of speech exchanged while teachers and children are wearing the hardware and moving through their day in the classroom. Once valid segments of speech are identified, the processing pipeline moves to stage two.

Stage two of data processing consists of the diarization of valid speech data. Diarization is the process of identifying who spoke in each audio recording. The system implements two-step diarization comprising (1) speech segmentation and (2) speaker classification. For speech segmentation, including overlapped speech detection, we fine-tuned Pyannote-audio (Bredin et al., 2020), using 120 min of manually timestamped classroom audio to identify whether a given utterance belongs to a teacher, a peer, or to the person wearing the recorder (focal child or focal teacher).

Stage three of data processing is transcription. The audio segments that contain speech are then processed via Whisper automatic speech recognition (ASR) (Radford et al., 2023), which produces timestamped, automatic transcripts. Stage four consists of text processing. During this stage transcripts are re-segmented using punctuation information returned by Whisper, as detailed in Gonzalez Villasanti et al. (2020), in order to approximate the SALT protocol for segmenting into C-units. A C-unit comprises one independent clause and all associated dependent clauses and modifiers. To approximate utterances, we segmented utterances using the punctuation marks returned by Whisper ASR as boundaries in accordance with earlier work using recordings of preschool classroom language environments (Gonzalez Villasanti et al., 2020).

In stage five, each utterance is matched with the proximity data to identify the participants in proximity of the focal person when utterances were spoken. The final stage consists of calculating interaction metrics at the dyadic level (sender-receiver). These metrics include the following: number of auxiliary verbs, number of coordinating conjunctions, number of adjectives, number of unique words, number of rare words, number of subordinating conjunctions, number of utterances (using Whisper punctuation as boundaries), number of verbs, number of words, mean length of utterance, type token ratio, conversational turns, and speech duration. To our knowledge, no other sensing system provides all such language measures. IDEAS offers a viable method for supplementing traditional methods for studying classroom environments.

### Sample

The study for which these data were collected is approved by the Institutional Review Board at The Ohio State University. The sample comprises 22 speakers (three classroom teachers and 19 children) who were a subset of the participant sample in a larger study (Chaparro-Moreno et al., 2019), which examined a classroom social network over a two-week period. The larger study collected 664 min of audio and video recordings in one preschool classroom in an urban early-learning center.

In the larger study, research staff solicited informed consent from all teachers (*n* = 3) and caregivers of children (*n* = 20) in the classroom. Consent procedures sought agreement for each participant to wear a head-mounted camera. All three classroom teachers consented. The three teacher participants reported their sex as female, and their level of education as an associate's degree or higher.

Of the 20 children in the classroom, 15 consented to wearing a head-mounted camera with audio-recording capability, whereas four had permission to be in the classroom and be recorded, but not wear the recording hardware. The child who did not have permission to participate was moved to another classroom during the recording sessions per the recommendation of the center director. The 15 consented children who wore the recording hardware were 47 months old on average (range = 35–58 months) and included 10 boys and five girls. Caregivers completed an initial family background questionnaire at the time of consent to provide basic demographic information for the study. The children were relatively diverse with 67% of caregivers reporting their child's race as African American, 27% as white, and 6% as un-reported or another race. In terms of highest level of maternal education, 5% reported not completing high school, 5% completed high school, 26% completed a certificated training after high school, 21% completed a bachelor's degree, and 37% reported having obtained a graduate degree. Of note, 6% of caregivers did not report the maternal level of education for their household.

### Procedures

In fall of the academic year, the fully consented participating children wore a head-mounted GoPro camera with an audio recording feature over a one-week period for a total of 664 min of recordings; for each child, between 36 and 59 min of recordings were collected. Each participating child wore the GoPro camera on one randomly assigned day for 1 h during the morning and 1 h during the afternoon. Each day, four children wore the camera simultaneously for the purposes of capturing both children's own talk and peer talk to children. Prior to data collection, classroom teachers and research staff piloted the cameras over a one-week period prior to them being worn by the children. The purpose of this piloting work was to assess comfort and determine the battery life for the devices. The research team determined that the camera should be worn by children for 1 h maximum in a given session because it became warm and could potentially cause discomfort. Data used for this study is from the morning session. The rationale for using data from the morning session was that during this period, children experienced varied activities (e.g., free choice, whole group, transitions), which represent common contextual factors associated with preschool classroom settings. The data we used in these specific analyses was recorded over three consecutive days. In day one, only one child recording was used. On day two, recordings from three children were used. On day three, recordings from two children were. Given that there were 19 children in the classroom each day, it is possible but not likely that the talk from a child wearing the sensor is also captured by another child's sensor.

**Figure 2:**
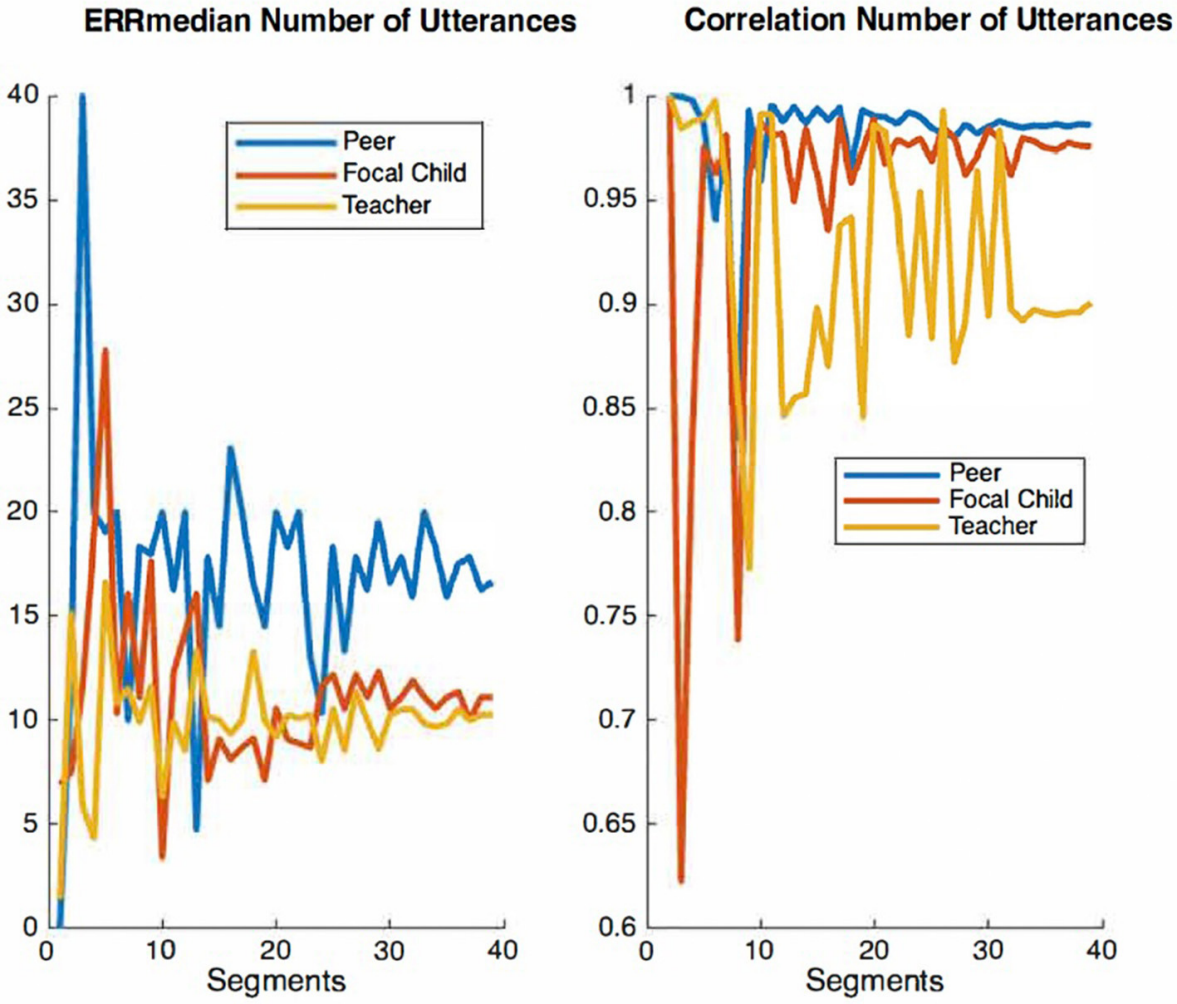
Convergence plots of ERRmedian and correlation values for number of words across audio segments.

**Figure 3:**
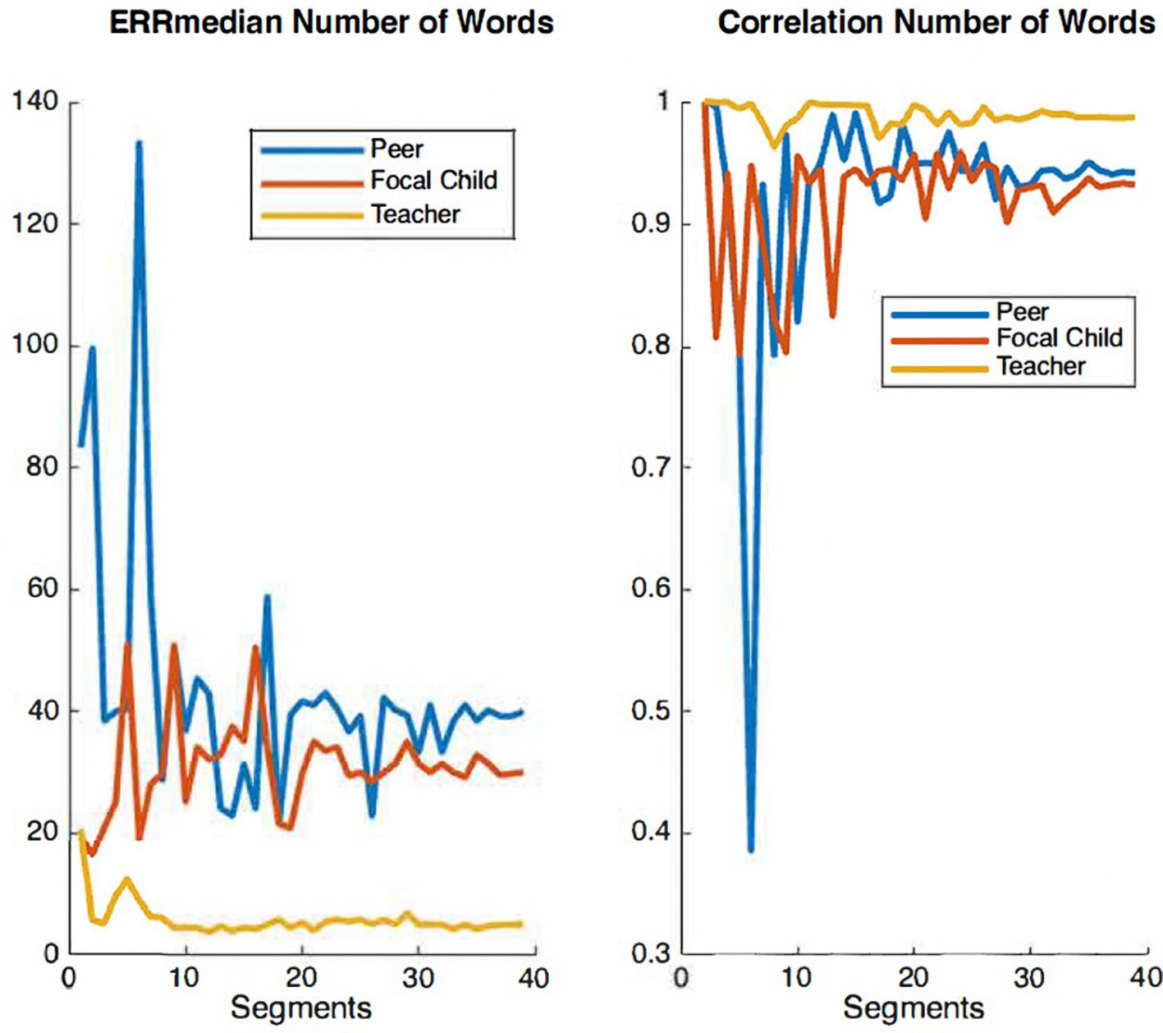
Convergence plots of ERRmedian and correlation values for number of nouns across audio segments.

**Figure 4:**
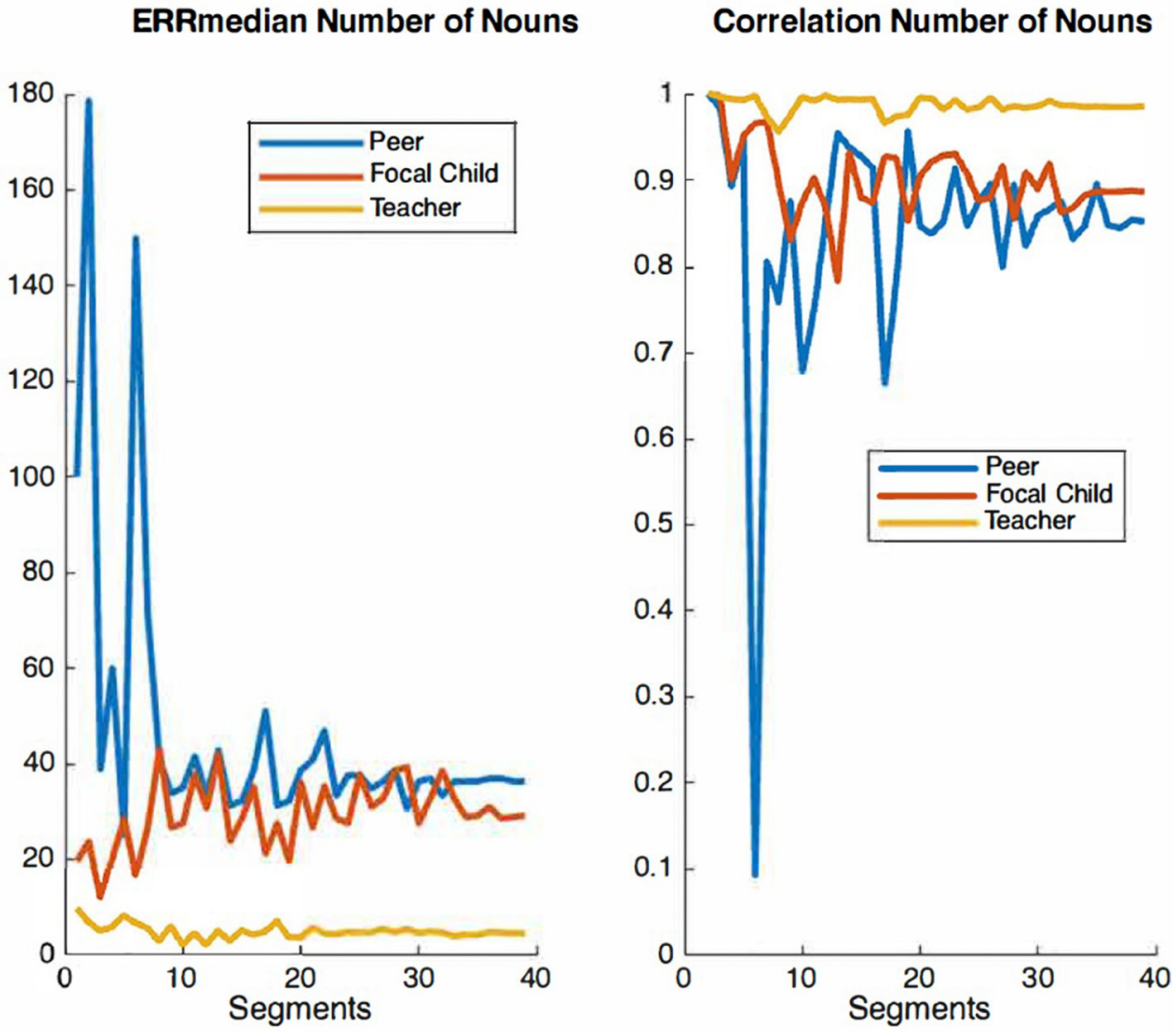
Diagram of IDEAS data pipeline.

For the present study, we elected to use the audio recordings of six children, which were manually timestamped and transcribed by trained observers for the purpose of examining IDEAS accuracy (Chaparro-Moreno et al., 2019). The segments were non-concurrent recordings randomly selected from 1 h recording sessions collected in the mornings over a two-week period. Our rationale for selecting six children was twofold. First, using the recordings of these six children allow for a larger corpus of peer-talk from children not wearing recorders directed to children wearing recorders. Second, there are extensive resource demands associated with timestamping and transcribing recordings using the conventions overviewed below. The time cost of manually timestamping and transcribing 10 min of child-speech audio is ~5 h. We therefore examined convergence plots for both *ERR*_median_ and correlation values across audio segments to ensure ample data are used for the analyses of system accuracy. Convergence plots are used to determine whether there is sufficient quantity of a given measure to evaluate its accuracy with precision. In a graph that displays acceptable convergence, the curve value becomes asymptomatic as its plot values increase along the x-axis. Figures 2–4 show convergence plots for number of words, number of utterances, and number of nouns. The coding team began to work with recordings of six children; the lead engineer monitored convergence. After the timestamped and transcribed recordings of six children were compared to those automatically transcribed and scored by IDEAS, curve values became sufficiently asymptomatic (non-stochastic), indicating that ample timestamped and transcribed data are used for these analyses of IDEAS system accuracy.

Given the focus on the accuracy of directed speech from both teachers and classmates, only talk identified as directed to the focal children (i.e., the six children wearing the recorder) was used for this study. Thus, the recording segments used as ground truth for this study represent “direct talk” from teachers and peers. Prior to transcription, coders identified valid audio segments containing direct talk in accordance with the scheme presented in Fernyhough and Russell (1997), which adhered to the following criteria: (a) teacher or peer behaviors involved the focal child while speaking (e.g., proximity and orientation), (b) teacher or peer speech was topically related to the focal child's preceding utterance, was a direct question, or contained the child's name, or (c) teachers or peer utterances occurred within 3 s of the focal child's utterance. The duration of talk from each speaker type was as follows: (a) teacher-directed talk to children wearing recorders: 47.42 min, (b) peer-directed talk from the child not wearing the recorder: 14.59 min, and (c) child's own talk: 29.70 min. A total of 91.71 minutes of direct talk was identified across the three speaker types, all of which were subsequently transcribed manually.

#### Human transcription and coding

The 91.71 min of identified talk were transcribed verbatim by trained researched assistants using the *Systematic Analysis of Language Transcripts* software (SALT Research Version 28) (Miller and Iglesias, 2008). Transcripts were parsed based on communication units (C-units). Segmentation of running speech into C-units, rather than utterances, utilizes syntactic information for parsing running speech into smaller discrete units. As previously noted, one C-unit consists of one independent clause and all dependent clauses and phrases. In instances where running speech occurred without a clause structure, these were segmented as if they were a C-unit (Chaparro-Moreno et al., 2019).

The transcribers took several measures to ensure the accuracy of transcription. First, the transcribers completed a comprehensive training that included studying relevant materials, including the SALT manual. Second, they completed a series of practice sessions. Their transcripts were compared to gold standard transcripts established for training videos for each of these sessions. Third, the transcribers were required to complete five test sessions, which were compared against primary codes created by the lab's lead transcriber. All transcripts used for this study were then checked line by line by the coding team lead for accuracy.

When transcription was complete, 13 language indices were calculated for each speaker type using MATLAB's natural language processing tool, the Text Analytics Toolbox, to process the text from both transcripts done manually by humans and those automatically transcribed using Whisper ASR. The tool breaks the text into smaller units called tokens (e.g., words), and then assigns labels to each token, such as verbs, adjectives, nouns, etc. Table 1 provides details on these 13 indices. In sum, ground truth for this study is defined as: human diarization and transcription with no automatic resegmentation performed. Calculation of linguistic measures is then conducted on the manually diarized and transcribed data using MATLAB's text analytics toolbox based on the manually diarized human transcription.

**Table 1:** Abbreviations, brief definitions, and examples for linguistic indices in IDEAS.

**Linguistic indices**	**Abbreviation**	**Definition**	**Example**
Number of utterances	UT	Total count of utterances occurring in a given recording, automatically labeled by IDEAS after a pause <0.3 s or if a punctuation mark was detected in the transcript.	
Number of words	WD	Total count of words (non-punctuation units), a singular element of meaningful speech, used either in isolation or in combination with others to form a sentence, automatically captured by IDEAS in the transcript.	
Number of auxiliary verbs	AV	Total count of auxiliaries that accompany the lexical verb of a verb phrase for grammatical distinctions and copula BE.	I *am* going to read you something off of my cards; You *are* right.
Number of coordinating conjunctions	CC	Total number of coordinating conjunctions, words that link words or larger constituents without syntactically subordinating one to the other and express a semantic relationship between them.	and, or, but; I do not know if I have green scissors, *but I have pink ones*.
Number of subordinating conjunctions	SC	Total number of subordinating conjunctions, words that link constructions by making one of them a constituent of the other.	that, if, while; You can use the mirror *if* you need it.
Number of verbs	VB	Total number of main verbs (content verbs) that typically signal events and actions.	jump; laugh
Number of adjectives	AJ	Total number of adjectives, words that typically modify nouns and specify their properties or attributes.	good; little; tall
Number of unique words	UW	Total count of words that appeared in a given recording at least once, automatically captured by IDEAS.	
Number of rare words	RW	Total count of rare words in a given recording, automatically captured by IDEAS. Rare words are defined in accordance with the scheme established by Hayes and Ahrens (1988), excluded from the list of the 10,000 most common words and their inflected forms, and is not a proper name or number.	
Mean Length of utterance	MLU	A measure of linguistic productivity calculated by dividing the number of words by the number of utterances.	
Type token ratio	TTR	A measure of linguistic complexity in vocabulary calculated by dividing the total number of unique words (types) by the total number of words (tokens).	
Number of conversational turns	CT	Number of conversational turns is the total count of back-and-forth alternations between speakers in a given recording.	
Speech duration	SD	The total duration that a given speaker spoke during a recording in seconds.	

Definitions for AV, CC, SC, VB, and AJ are provided by Universal Dependences (https://universaldependencies.org/), the annotation scheme used in Matlab's text analytic toolbox (The Mathworks Inc., 2020). Examples are extracted from contexts in the transcripts returned by Amazon Transcribe speech-to-text service.

For details of IDEAS automation, see Gonzalez Villasanti et al. (2020).

#### IDEAS automated transcription and coding

For the present study, we used IDEAS to analyze the 91.71 min of teacher-directed talk, peer-directed talk, and child's own talk to evaluate the system's accuracy for calculating the 13 language indices following its automated transcription feature. Here, we describe the IDEAS pipeline in terms of transcription and calculating linguistic indices.

First, the entire recording for each participant was timestamped manually as described previously. A randomly selected 10% of clips were double coded to ensure the accuracy and monitor drift. This process entailed noting the offset and onset time of each speaker using the ELAN software (EUDICO Linguistic Annotator; The Language Archive, 2022). Any segments falling within the established threshold of 250-milliseconds of demarcated speaker type by both coders were considered in agreement, whereas any onset of offset times between transcribers exceeding the 250-millisecond threshold were deemed not in agreement. The percent of absolute agreement across coders for onset coding was 86.52. The percent of absolute agreement across coders for offset coding was 84.44. Although the most updated IDEAS feature set includes automatized timestamping (i.e., diarization), the present study involved manual timestamping because existing diarization algorithms were not accurate with our dataset due to the insufficient audio quality caused by collecting these initial recordings via GoPro. We acknowledge this as a potential limitation of the current study.

Second, the timestamped audio segments containing valid speech were processed via the Whisper automatic speech recognition system, which returns automatically generated transcripts. Whisper is an open-source, automatic speech recognition tool recently developed and freely available for use.

Third, the MATLAB program subsequently re-segmented transcripts returned by Whisper using punctuation information, as detailed in Gonzalez Villasanti et al. (2020), in order to approximate the SALT protocol for segmenting in C-units. The program then calculated the linguistic indices for each speaker type (see Table 1). In sum, test data used for comparison to ground truth in this study is defined as: human diarization followed by automatic transcription using Whisper ASR. Whisper-generated transcripts are then automatically resegmented using MATLAB in accordance with the scheme developed by Gonzalez Villasanti et al. (2020). Indices are subsequently calculated using MATLAB's text analytics toolbox. It should be noted that manually timestamping data prior to using the automatic features comprising the IDEAS pipeline as compared to using the fully automated feature set that includes automatic timestamping could result in inflated accuracy metrics for the 13 indices reported in the results section of this manuscript. However, using this same method on recordings of poor audio quality such as those collected via GoPro for this study could result in similar accuracy outcomes and could offer substantial time saving to researchers.

### Accuracy analysis: *ERR*_*median*_, correlations, and MWER

To conduct the accuracy analysis for IDEAS, all teacher and child audio segments were parsed into six-minute segments. Our rationale for this segmentation process was to normalize the recording duration for which each set of accuracy metrics were calculated. Using this shorter segment duration also allows for examining correlations for each segment between manually transcribed and automatically transcribed data. This segmentation process is a conservative approach, as using longer segments such as 30 min or 1 h would result in inflated accuracy rates.

Two metrics were calculated on each of these six-minute segments by comparing correlation coefficients and *ERR*_median_ for IDEAS relative to ground truth across the following three constructs: (a) teacher talk to children, (b) peer talk to children, and (c) children's own talk. Thus across each of the three constructs, for a given language index *X* , we collect the vectors *X*_*p*_ = [*X*_*p*, 1_, …, *X*_*p, n*_] and *X*_*r*_ = [*X*_*r*, 1_, …, *X*_*r, n*_], where *X*_*r, j*_ and *X*_*p, j*_ are the ground truth (reference) and predicted measures for segment *j* . We compute the linear correlation (R) between *X*_*p*_ and *X*_*r*_, and the *ERR*_median_ across segments for each speaker type across the thirteen indices.

Correlation coefficients represent the size and nature of the relationship between two constructs, whereas *ERR*_median_ is median of the relative absolute errors of segments, where the relative absolute error is the absolute difference between IDEAS-predicted feature value and ground-truth feature value for the segment, divided by the ground-truth feature value. We calculated the absolute relative error for each of the 13 linguistic indices on each of the six-minute recording segments and report the median of the absolute relative error across all segments. For a segment *j* , the absolute relative error, represented by *E*_*X, j*_≥0, is computed by measuring the deviation between the values obtained by using the manual transcription *X*_*r, j*_ and predicted transcripts *X*_*p, j*_ by


(1) $$\begin{array}{l} E_{X,j}=\frac{\left|X_{r,j}-X_{p,j}\right|}{X_{r,j}}\cdot 100 \end{array}$$

with values close to zero representing less deviation from the reference values. Our rationale for using *ERR*_median_ is threefold. First, it has been used in a relatively recent study to examine the accuracy of the open-source Automatic Linguistic Unit Count Estimator (ALICE) system (see Räsänen et al., 2020). Second, while researchers examining the accuracy of automatic speech recognition systems may more frequently report the mean absolute relative error (Cristia et al., 2020; Ferjan Ramírez et al., 2021), we opted to use ERRmedian given the literature examining the accuracy of these systems that argues for the use of the median instead of the mean to account for the fact that the absolute relative error distribution can be highly skewed (Räsänen et al., 2020). To that effect, we examined the normality distribution of absolute relative errors across the 13 indices for each speaker type (teacher talk, peer talk to children, children's own talk). Histograms indicated that data was skewed and contained outliers across all measures. Normality of the error distributions was further investigated with Kolmogorov-Smirnov test. Test results indicated a non-normal distribution of errors. We therefore chose the median as it is more robust to outliers and skewed distributions in alignment with the approach used by Räsänen et al (2021) when evaluating the accuracy of a similar system. Third, using the median prevents the nullification of under and overestimates made by the system.

Whereas the correlation coefficients and *ERR*_median_ are used to evaluate overall accuracy for a given speaker type across all 13 linguistic indices, we also calculated median word error rate (MWER). MWER was used to assess the overall accuracy of IDEAS for a given speaker type across all recording segments comprising a data set. To measure the MWER for IDEAS, we calculated the word error rate for each of the six-minute recording segments comprising each speaker type and report the overall median value across all recording segments for a given speaker type. To our knowledge, this manuscript is one of the first to report MWER for the automated transcription and scoring of recordings collected in a naturalistic preschool setting.

## Results

The primary aim of this study was to examine the accuracy of IDEAS in terms of capturing children's language environments with reference to three constructs: (a) teacher talk to a child, (b) peer talk to a child, and (c) child own talk. To address this aim, we compared IDEAS' outcomes calculated via manually timestamped and automatically transcribed data relative to those calculated via manually timestamped and transcribed data, which served as ground truth for this study, collected within a preschool classroom. Tables 2–4 summarize the *ERR*_median_ and linear correlation for each speaker type and the 13 linguistic indices, with interpretation provided in the next section. Note that Tables 2–4 also provide estimate totals for ground truth (Sum GT) and IDEAS (Sum IDEAS). These estimate totals provide descriptive data on the number of instances each linguistic index was coded by ground-truth (i.e., MATLAB text analytics toolbox coding manually timestamped and transcribed data) and IDEAS.

**Table 2:** Accuracy of IDEAS vs. ground truth for teacher talk.

**Measure**	***ERR*_median_**	**R**	**Sum GT**	**Sum IDEAS**
Auxiliary verbs	7.42	0.96^**^	818.00	819.00
Coordinating conjunctions	14.29	0.95^**^	191.00	180.00
Adjectives	16.67	0.95^**^	397.00	345.00
Unique words	4.52	0.99^**^	3,335.00	3,211.00
Rare words	25.00	0.89^**^	366.00	289.00
Subordinating conjunctions	0.00	0.89^**^	31.00	27.00
Utterances	10.26	0.90^**^	1,407.00	1,309.00
Verbs	10.13	0.98^**^	710.00	688.00
Words	5.01	0.99^**^	8,305.00	7,906.00
Mean length of utterance	4.04	0.95^**^	17.63	17.85
Type token ratio	11.37	0.81^**^	211.99	212.02
Conversational turns	5.00	0.98^**^	316.00	329.00
Speech duration	9.72	0.99^**^	2,738.60	2,827.30

ERR_median_, Median Absolute Relative Error; R, correlation coefficient; Sum GT, total units detected by transcribers; Sum_IDEAS, total units detected by IDEAS.

^*^ <0.05; ^**^ <0.01.

**Table 3:** Accuracy of IDEAS vs. ground truth for peer talk to children.

**Measure**	***ERR*_median_**	**R**	**Sum GT**	**Sum IDEAS**
Auxiliary verbs	50.00	0.70^**^	81.00	125.00
Coordinating conjunctions	100.00	0.53^**^	8.00	16.00
Adjectives	40.00	0.76^**^	59.00	72.00
Unique words	36.36	0.85^**^	741.00	881.00
Rare words	62.35	0.65^**^	80.00	69.00
Subordinating conjunctions	50.00	0.86^**^	7.00	3.00
Utterances	16.67	0.99^**^	462.00	521.00
Verbs	46.43	0.85^**^	111.00	121.00
Words	40.00	0.94^**^	1,327.00	1,644.00
Mean length of utterance	12.00	0.65^**^	23.52	23.45
Type token ratio	24.14	0.36^*^	98.17	119.99
Conversational turns	0.00	0.98^**^	197.00	210.00
Speech duration	22.61	0.99	709.56	866.68

ERR_median_, Median Absolute Relative Error; R, correlation coefficient; Sum GT, total units detected by transcribers; Sum_IDEAS, total units detected by IDEAS.

^*^ <0.05; ^**^ <0.01.

**Table 4:** Accuracy of IDEAS vs. ground truth for children's own talk.

**Measure**	***ERR*_median_**	**R**	**Sum GT**	**Sum IDEAS**
Auxiliary Verbs	55.56	0.79^**^	160.00	277.00
Coordinating Conjunctions	50.00	0.50^**^	30.00	41.00
Adjectives	37.50	0.91^**^	133.00	143.00
Unique Words	29.17	0.89^**^	1,370.00	1,703.00
Rare Words	40.00	0.61^**^	158.00	149.00
Subordinating Conjunctions	100.00	0.71^**^	3.00	6.00
Utterances	11.11	0.98^**^	949.00	1,026.00
Verbs	33.33	0.84^**^	179.00	236.00
Words	30.00	0.93^**^	2,770.00	3,470.00
Mean Length of Utterance	9.29	0.27	21.30	21.04
Type Token Ratio	17.43	0.26	114.39	132.93
Conversational Turns	0.00	0.98^**^	398.00	407.00
Speech Duration	20.21	0.99^**^	1,487.00	1,774.90

ERR_median_, Median Absolute Relative Error; R, correlation coefficient; Sum GT, total units detected by transcribers; Sum_IDEAS, total units detected by IDEAS.

^*^ <0.05; ^**^ <0.01.

### Teacher-directed talk to children

Measurement of teacher-directed talk was captured via the recorders worn by children on which teacher speech was detected. Correlations, *ERR*_median_ results, and descriptive findings for teacher-directed talk are shown in Table 2. With respect to correlations between IDEAS and ground-truth for this construct, correlation coefficients ranged from *r* = 0.81 (type token ratio) to *r* = 0.99 (number of words, speech duration, number of unique words). All were statistically significant (*p* < 0.05), and can be interpreted as highly correlated (Hemphill, 2003).

For *ERR*_median_, values ranged from 25.00 (rare words) to 0.00 (subordinating conjunctions). The closer *ERR*_median_ is to 0, the more accurate the model is for a given index. Of note, IDEAS detected 1,309 of the 1,407 utterances identified in the ground truth data. For number of words, IDEAS detected 7,906 of the 8,305 units identified in the ground truth teacher-directed talk data.

### Peer talk to children

Measurement of peer talk to children was captured via the recorders worn by the six children included in the children's own talk corpus on which speech from other peers was detected. Correlations, *ERR*_median_ results, and descriptives for peer-directed talk are displayed in Table 3. Correlation coefficients between IDEAS and ground-truth ranged from *r* = 0.36 (type token ratio) to *r* = 0.99 (number of utterances, speech duration). All were statistically significant (*p* < 0.05) and can be interpreted as medium-sized or larger in magnitude (Hemphill, 2003).

*ERR*_median_ for peer talk to children ranged from 100.00 (coordinating conjunctions) to 0.00 (conversational turns). Several indices showed IDEAS *ERR*_median_ below 30. These include type token ratio, speech duration, number of utterances, and mean length of utterance, and conversational turns. Although the *ERR*_median_ for coordinating conjunctions was 100, there were only 8 units identified by manual transcribers in the entirety of the peer talk recordings, for which IDEAS overestimated by identifying 16. For number of utterances, although there were 462 identified by manual transcribers, IDEAS detected 521. For number of words, IDEAS detected 1,644, whereas 1,327 of these units were identified in the ground truth data of peer talk to children wearing recorders.

### Children's own talk

Given our focus on recording children in the preschool classroom context, focal child speech is of primary interest for this study. Children's own talk was captured via the recorders worn directly by children speaking. Correlations, *ERR*_median_ results, and descriptive findings for children's own talk are shown in Table 4. Regarding correlations between IDEAS and ground-truth manual coding, correlation coefficients ranged from *r* = 0.26 (type token ratio) to *r* = 0.99 (speech duration). All but two were statistically significant (*p* < 0.05) and can be interpreted as ranging from low to high (Hemphill, 2003). Interestingly, there were two indices (rate of number of unique words per utterance and rate of number of words per utterance) that did not have significant correlations despite these being highly correlated for the speech of peers to children wearing recorders. One possible explanation for this is the fact that there was a shorter corpus of recordings available for peer talk.

*ERR*_median_ for child's own talk ranged from 100.00 (subordinating conjunctions) to 0.00 (conversational turns). Several indices showed error rates at or below 30 (number of words, number of unique words, speech duration, type token ratio, number of utterances, mean length of utterance, and conversational turns). Although *ERR*_median_ for subordinating conjunctions was 100, there were only three units identified in the ground truth data, of which IDEAS detected six. There were 949 utterances for child's own talk identified by MATLAB in the ground truth data, IDEAS detected 1,026. With respect to number of words, IDEAS detected 3,470, whereas a total of 2,770 were detected in the ground truth child's own talk data.

### Median word error rate (MWER)

Median Word Error Rate (MWER) is indicated for each speaker type in Table 5. IDEAS had the highest MWER for peer talk to children wearing recorders, followed by children's own talk. MWER was lowest for teacher talk. Our findings align with the limited other studies of automated speech processing systems, which show higher error rates for child speech than the speech of adults (Lee et al., 1997; Potamianos et al., 1997; Chaparro-Moreno et al., 2023).

**Table 5:** IDEAS median word error rate for each speaker type.

**Speaker type**	**MWER**
Teacher-talk to children	30.41
Peer-talk to children	73.77
Child's own talk	62.75

MWER, median word error rate.

## Discussion

Understanding preschool language environments is of keen interest to educational and developmental researchers. Recent work employing sensing technologies demonstrates the feasibility of using audio recorders in preschool settings (Gonzalez Villasanti et al., 2020), and these have improved understanding of classroom language experiences that influence children's language development and language-learning opportunities (Ferguson et al., 2020; Choi et al., 2023). However, there is a need to address limitations of the available sensing technologies, including measurement limitations (e.g., limited breadth of language experience measures provided) and high costs. The goal of this study was to examine the accuracy of IDEAS in terms of capturing children's language environments with reference to three constructs: (a) teacher talk to children, (b) peer talk to children, and (c) children's own talk. Across each of these three constructs, we measured accuracy of thirteen automated indices of teacher, peer, and children's own talk as shown in Tables 2–4. This study makes an important contribution to the literature, because no other sensing systems of which we are aware used for the study of language environments in naturalistic preschool settings provide this range of output measures.

Here we report initial findings on the accuracy of the system's measures of teacher and child talk collected in a naturalistic preschool setting. IDEAS provides a means by which to affordably capture day-long recordings of proximity and linguistic environments across a variety of contexts including preschool classrooms. While cost depends on the corpus of data being collected, some scholars point to the prohibitively expensive nature of using LENA (Bang et al., 2022). For instance, to provide four children each a LENA recorder, the total cost of hardware would be $1,649, as compared to $375 for the standard Sony recorders typically used for IDEAS recordings. Additionally, data can be processed through the IDEAS pipeline for the cost of a research assistant's hourly rate. However, LENA requires one to solicit a quote from the LENA company and then pay based upon the volume and duration of recordings being collected and processed. Affordably capturing accurate estimates of the language children experience is useful to outlining the linguistic exposure necessary in supporting language learning. For instance, with data from multiple classroom recordings using IDEAS, one could use utterance complexity as a measure to predict children's longitudinal language outcomes (Huttenlocher et al., 2002; Vasilyeva et al., 2008).

The primary findings of this study are: (a) statistically significant correlations between IDEAS automatically scored and ground truth data across all indices of teacher talk; (b) statistically significant correlations between IDEAS automatically scored and ground truth data across the majority of indices of peer and children's own talk; and (c) acceptable MWER for recordings of naturalistic preschool classroom environments across the adult and child speaker types.

Findings suggest IDEAS may be an accurate automated tool for providing a variety of indices of teacher talk using recordings collected in classroom settings. Further testing of the entire automated pipeline is needed, particularly given that data for both ground truth and IDEAS were manually timestamped prior to transcription.

With respect to child talk (peer-directed or child's own), for those indices with high magnitude correlation coefficients (e.g., number of utterances, number of words, conversational turns, speech duration), IDEAS may be used to accurately examine change in rates of linguistic output or input over time. However further refinement is needed before IDEAS can accurately measure child speech at a single time point across all thirteen indices with high levels of accuracy.

### Accuracy of IDEAS: teacher talk

Given that teacher speech used for this study was transcribed from recorders worn by children, the accuracy of IDEAS for classifying directed adult speech in classroom settings shows promise for future use. In quieter naturalistic settings, such as museums, word error rates for adult speech are commonly around 20% (Saggese et al., 2019). Considering that IDEAS using the Whisper small model was able to obtain an overall MWER of 30.41 in a noisy classroom environment without teacher subjects wearing the recording hardware, and the high magnitude correlations between IDEAS and ground truth across all indices, IDEAS can be used to measure teacher speech in preschool settings with relatively high precision.

The implications of these findings are that for a fraction of the price of using other language sensing systems (e.g., LENA), one could use IDEAS to accurately collect and process recordings of teacher talk in naturalistic preschool settings. Given the body of research indicating that high-quality interactions between teachers and children are positively associated with a range of social and academic outcomes (Cadima et al., 2010; Irvin et al., 2013; Langeloo et al., 2019), a tool that accurately provides a more robust set of indices on adult's output in children's naturalistic language environments is needed. While LENA measures such as adult word count and conversational turns are useful, additional indices would allow us to gain a more nuanced understanding of adult/teacher language output and in particular, the specific mechanisms of language exposure and exchange that may drive children's development over time.

### Accuracy of IDEAS: child talk

This study constitutes the first in which automated speech *ERR*_median_ for children in preschool classrooms are reported for IDEAS. For child speech collected in this setting, IDEAS shows commendable accuracy across several measures of interest such as number of words, number of utterances, and conversational turns. Nine of the 13 indices automated transcriptions of peer talk to children and children's own talk show correlations with ground truth data between the range of 0.70–0.99. However, four of the 13 indices (coordinating conjunctions, rare words, mean length of utterance, and type token ratio) show correlations between indices calculated via automated transcription and ground truth estimates below the 0.70 threshold. Although the correlation values for Mean Length of Utterance for peer talk to children (0.65) and children's own talk (0.27) were lower than the 0.70 accuracy threshold, the median absolute relative error for both were lower than 20%, which meets the alternative accuracy criterion established by automatic speech recognition researchers (Räsänen et al., 2020). Nonetheless, further improvement of the automatic calculation of these measures may be needed before they can be used to accurately characterize children's speech in noisy classroom environments. These results may be due to several factors. First, the relatively small volume of data used for this study resulted in fewer instances in which these variables were present. Second, children wore an earlier iteration of the IDEAS hardware for this study. That is, they wore GoPro recorders in lieu of the Sony recorders typically used with IDEAS. Future data collected using our updated recorders and vest configuration may result in higher levels of accuracy for these indices of both peer and children's own talk.

While *ERR*_median_ is high for several indices, correlations are statistically significant and of high magnitude across many of the language indices provided by the system. This means that IDEAS may be used to accurately measure change over multiple observation time points both between and within subjects.

There is a salient need for IDEAS and developers of automated speech processing systems in general to reduce error rates for child speech. There is also a need for researchers to provide transparency for the research community regarding the accuracy and constraints of automated language sensing systems across a variety of contexts. Most studies conducted using language sensing in naturalistic preschool settings examine research questions relating to the language input of adults directed to the child, often in one-on-one settings (Greenwood et al., 2018). This work seeks to fill a critical gap by designing a sensing system that can be used to gather objective data on the natural language experiences of both teacher *and* children in early childhood classroom settings. Understanding the mechanisms by which teachers and peers support the development of other children has the potential to shape future classroom practice. These sensing approaches can further inform how we allocate resources to support the development of all children across a variety of contexts.

The typical method for studying teacher and child language experiences in preschool classrooms is in-person observations. Given that these methods are prohibitively costly in nature, researchers are often forced to rely on a single or infrequent observation made of a snapshot of the school day as their primary means for measuring linguistic interactions within classrooms. This may be problematic, as we know that language exchange in these settings may vary by activity type, location in the classroom, and time of day. Further, over the past five decades, the educational research community has expressed various concerns regarding the limitations associated with in-person observations. While sensing systems such as IDEAS may need further refinement on some measures of interest related to language exchange in classroom settings, they pose a possible solution for mitigating the barriers identified with the use of in-person observations including cost, bias, and the ability to observe the interactions of multiple subjects within an environment simultaneously.

A notable benefit of using these sensing tools is that they have the potential to shift us away from a deficit model of education. While such systems can help identify children with language learning needs, toward which critical teacher and peer language resources can be directed, they can also provide an important opportunity to learn more about all children in the classroom, without the potentially disruptive presence of in-person observers. With systems such as IDEAS, researchers and practitioners are able to gain a more nuanced understanding of what kids *can* do, in a naturalistic environment, while interacting with peers and teachers over time.

The broader implications of these initial findings on the accuracy of IDEAS show that at minimum, one could collect and timestamp classroom recordings before using the IDEAS pipeline to automatically transcribe and analyze said recordings. IDEAS would provide the thirteen language indices reported in this manuscript. One could feasibly use any of the indices across speaker type with high correlation values, particularly when examining change over time. This process alone would save researchers or practitioners substantial cost and time resources required with human transcription. For instance, the approximate time cost associated with transcribing (not time stamping) a 10 min segment of audio using the SALT system overviewed in the methods section above averaged over 4 h. Thus, to transcribe and obtain output measures for 1 h of recording on each child in a classroom comprised of 18 children would require 432 h of transcription time. Many researchers and practitioners do not have the time and resources available to commit to this process. IDEAS processed these data in 6 h using a standard laptop, providing an alternative method for obtaining these language measures with substantial time cost savings to end users. It is also worth noting however that the full IDEAS feature set includes automatic timestamping of recordings providing that audio is of sufficient quality.

In our current work, we are deploying the IDEAS system across 30 early childhood classrooms, which will result in an estimated 1,000 h of classroom observations wherein all consented children in each classroom wear both recorders and beacons. These data will be used to conduct a more robust validation of the IDEAS automated speech classification system and the system's broader feature set while using a more effective audio recording device. This ongoing work will allow our team to further examine the accuracy and potential of IDEAS, which is a necessary step in preparation for future scaling.

### Limitations and opportunities

This study has several noteworthy limitations. Though IDEAS was designed to address the unique challenges of automatic language transcription of young children in preschool classroom environments, some problems persist. First, there are complications with recording many children simultaneously. Classrooms are noisy environments with concurrent activities and overlapping conversations. This is further complicated by the imperfect nature of the speech of young children whose language skills are still developing. While sensing technologies and the findings overviewed in this manuscript show promise, further refinement is needed before we can shift to providing *in vivo* feedback to practitioners with confidence. Second, the ground-truth values used in this study are based on an automatic tool (the MATLAB NLP toolbox) in lieu of having an expert (i.e., a linguist) define the feature values for manually transcribed data. It is likely the MATLAB NLP toolbox does a sufficient job of extracting the measures of interest; however, some small differences may exist between fully manual and semi-automated “gold standard” data presented in this manuscript.

Several opportunities for refinement of automatic speech recognition in early childhood classroom environments are currently being explored by our research group. First, it is worth examining the extent to which signal-to-noise ratios may vary depending on the level of background noise across classroom activity settings. This would allow researchers to identify best practices for conducting classroom recordings to optimize audio quality. However, we would caution against developing a one-size-fits-all approach given the variability of preschool classroom language environments. Second, multiple children wearing recorders provides opportunities for further optimizing classroom recording audio quality. Understanding how we can sample from multiple children and teachers' recordings may result in higher levels of transcription accuracy as well as lower error rates for IDEAS indices. Lastly, the accuracy is automatic speech recognition software is rapidly improving. Exploring tools other than those used in our current pipeline as they are made available may further improve the results presented in this manuscript.

Most notably, until a larger volume of audio data are analyzed via the full IDEAS pipeline, validation metrics and results should be interpreted with relative caution. Recent validation work by Räsänen et al. (2020) used a corpus of manually annotated recordings comprising 36.5 total hours. Subsequent work will report validation findings with IDEAS using data of a comparable scale. Additionally, future validation work would benefit from using the automatic diarization feature of IDEAS as opposed to manually timestamping data before using the component of the pipeline that incorporates Whisper automatic transcription. This would mitigate the potential inflation of IDEAS generated indices as result of data first being manually diarized.

An additional limitation worth noting is the data presented herein are from a classroom comprised of native English speakers. A fundamental challenge with automated language transcription and analysis systems is that accurate word count estimation of a particular language necessitates expertise in the language's phonology and lexicon. Incorporating this vast amount of data into the system virtually all the world's languages is not feasible (Räsänen et al., 2020). This is particularly true for less common languages for which transcribed data is sparse. There is a salient need for IDEAS and naturalistic sensing systems more broadly to be utilized in more diverse and underrepresented cultural linguistic contexts. Relatedly, the performance of the tools incorporated into the IDEAS pipeline (i.e., Whisper and the MATLAB NLP toolbox) are not equally accurate across all languages, therefore the accuracy of the IDEAS pipeline is likely to vary depending on the language in recordings being processed.

We would also like to note broader limitations to the use of sensing systems such as IDEAS, LENA, and Ubisense, among others. First, these systems do not capture data on the nonverbal aspects of communication, which are often essential for understanding the valence of interactions. In many instances in classrooms and across other contexts, communication occurs nonverbally via gestures, facial expressions, and the like (Ahuja et al., 2019). Second, these systems provide limited data on the qualitative aspects of speech. Although speech complexity could be examined from a number of measures provided by IDEAS, advances are required before these systems can provide accurate information on the qualitative nature of interactions in the classrooms or identify specific words exchanged between speakers. Further work is needed to expand the capabilities of these systems for analyzing additional features of classroom environments. It is the opinion of the authors that sensing systems should supplement, not replace expert observations.

Future validation work using IDEAS could benefit from examining adult-child dyadic interactions outside of classroom settings, as a large number of studies using similar technologies use data of this kind (Xu et al., 2009; Räsänen et al., 2020). While the intent of the IDEAS system was for use in naturalistic preschool settings, this additional data point would provide a more holistic picture of the system's capabilities and future potential across contexts.

## Data availability statement

The data analyzed in this study is subject to the following licenses/restrictions: IRB approved study team members may access data. Code for IDEAS is freely available for use. Requests to access these datasets should be directed to GP, pelfrey.19@osu.edu.

## Ethics statement

The studies involving humans were approved by The Ohio State University Social and Behavioral Institutional Review Board. The studies were conducted in accordance with the local legislation and institutional requirements. Written informed consent for participation in this study was provided by the participants' legal guardians/next of kin.

## Author contributions

GP: Conceptualization, Methodology, Writing – original draft, Writing – review and editing. LJ: Conceptualization, Data curation, Funding acquisition, Project administration, Resources, Supervision, Writing – review and editing. HG: Formal analysis, Methodology, Software, Visualization, Writing – review and editing. TF: Writing – review and editing.
